# High MICAL1 expression correlates with cancer progression and immune infiltration in renal clear cell carcinoma

**DOI:** 10.1186/s12885-022-10462-1

**Published:** 2022-12-27

**Authors:** Yixing Yang, Fengwen Ye, Tianxiang Xia, Qianwen Wang, Jun Du

**Affiliations:** 1 The First Clinical Medical College, Nanjing Medical University, Nanjing, 211166 China; 2 Department of Physiology, Nanjing Medical University, Nanjing, 211166 China

**Keywords:** MICAL1, Cell migration, Prognosis, Renal clear cell carcinoma, Immune infiltration

## Abstract

**Background:**

Molecule interacting with CasL 1 (MICAL1), a multidomain flavoprotein monooxygenase, is strongly involved in the biological processes related to cancer cell proliferation and metastasis. However, there were few reports on the clinical significance of MICAL1 in renal clear cell carcinoma.

**Methods:**

The expression and prognostic value of MICAL1 in renal clear cell carcinoma were explored using immunohistochemical assays, public TCGA-KIRC databases and multiple analysis methods, including survival analysis, univariate and multivariate analyses, KEGG and GSEA. Wound healing and Transwell assays were performed to check the 786-O cell and Caki-1 cell migration abilities after knockdown of MICAL1. Western blotting was used to assess the regulatory effect of MICAL1 on the Rac1 activation. Additionally, the function of MICAL1 and the correlations between MICAL1 and immune infiltration levels in KIRC were investigated using TIMER and TISIDB.

**Results:**

MICAL1 expression was significantly higher in carcinoma tissue compared with non-cancerous tissue. A survival analysis revealed that patients with high MICAL1 expression had shorter overall survival (OS) and disease-specific survival (DSS) compared with patients with low MICAL1 expression. ROC analysis also confirmed that MICAL1 has a high diagnostic value in KIRC. Importantly, the univariate and multivariate Cox analysis further confirmed that high MICAL1 expression was an independent risk factor for OS in patients with KIRC. In accordance with this, knockdown of MICAL1 expression decreased Rac1 activation and cell migration. KEGG and GSEA analysis revealed that the immune infiltration and Ras signaling pathways were significantly upregulated in the high MICAL1 expression group. In terms of immune infiltrating levels, MICAL1 expression was positively associated with CD8+/Treg cell infiltration levels. Specifically, bioinformatic analysis showed that MICAL1 expression had strong relationships with various T cell exhaustion markers.

**Conclusions:**

MICAL1 expression may act as a prognostic biomarker for determining the prognosis in renal clear cell carcinoma and plays an important role in regulating tumor immune microenvironment and cell migratory capacity.

**Supplementary Information:**

The online version contains supplementary material available at 10.1186/s12885-022-10462-1.

## Introduction

Renal cancer accounts for 2.2% of malignant tumors and usually affects adults (more males than females) that are approximately 70 years old [1]. Pathologically, most renal cancer is kidney renal clear cell carcinoma (KIRC), which accounts for 70–80%, while other forms, including kidney renal papillary cell carcinoma (KIRP), account for 7–14%, and kidney chromophobe (KICH) for 4–10%, etc. Patients may have no obvious clinical symptoms in the early stage, and the increase in tumor volume, hematuria, renal pain, and other discomforts were the most common signs. After surgical treatment, the 5-year survival rate of patients at the early stage of KIRC is more than 90%. However, the survival rate of patients at the advanced stage is only about 20%. Therefore, understanding the biological mechanisms of KIRC progression and developing effective molecular-based prognostic markers are of great importance.

The actin cytoskeleton is a complex, dynamic network of interlinking actin filaments present in the cytoplasm of cells. The disorder of the actin cytoskeleton will affect various fundamental cellular processes, including maintenance of the cell morphology, adhesion, migration, and invasion, and may further result in tumorigenesis [2]. Molecule interacting with CasL 1 (MICAL1), as a newly discovered actin cytoskeleton regulator, controls the terminal step of cell division by promoting F-actin depolymerization. MICAL1 has four conserved domains: an N-terminal flavin adenine dinucleotide (FAD) binding domain, a calponin homology (CH) domain, a Lin11, Isl-1 and Mec-3 (LIM) domain, and a C-terminal coiled-coil (CC) domain, where the FAD domain contains flavin monooxygenase activity and is responsible for ROS production of MICAL1 [3]. In the past years, studies have demonstrated that MICAL1 triggers F-actin disassembly and mediates vesicle unloading at the midbody [4]. MICAL1 is also shown to mediate the export of E-cadherin, MMP14, and CFTR DeltaF508 [5]. MICAL1 has been found overexpressed in several types of human cancers, including pancreatic adenocarcinoma and melanoma [6, 7]. Of note, increased oxidative stress was presented in many human metastatic tumors, and the roles of ROS in triggering signaling pathways for cell proliferation and invasion have been recognized for a long time [8]. So, combined with the characteristic of cargo transportation, recent evidence revealed the role of MICAL1 in carcinogenic biological processes, including cancer cell proliferation, invasion, and survival regulation [6, 7, 9, 10]. Recent study showed that MICAL1 acts as a activator for Rac1 activation which then promotes gastric cancer cell migration under hypoxia [11]. Rho GTPase effector PAK1 was also reported to associate with MICAL1 [12]. However, the role of MICAL1 in the prognosis of KIRC and its possible pathogenesis remains unknown. Therefore, by multiple databases, we explored the expression, prognosis, as well as tumor infiltrating lymphocytes of the MICAL1 in KIRC.

In this study, we used The Cancer Genome Atlas (TCGA) dataset and immunohistochemical assays to examine the expression of MICAL1 in KIRC tissue samples to determine its clinicopathological significance. In addition, we evaluated the role of MICAL1 in migration capacity of reanl cancer cells using functional assays. We further explore the possible cellular mechanism of MICAL1 through the Kyoto Encyclopedia of Genes and Genomes (KEGG), Gene Set Enrichment Analysis (GSEA), and Tumor IMmune Estimation Resource (TIMER), TISIDB analysis. This is the first comprehensive study of associations between the expression of MICAL1 and its clinical characteristics in KIRC and may help optimize immunotherapy for KIRC patients.

## Methods

### Ethics statement

All immunohistochemistry assays with human tumor specimens were conducted under the institutional guidelines of Jiangsu Province.

### Patients in the TCGA database

The gene expression of *MICAL1* in kidney carcinoma and corresponding clinical information data were downloaded from the TCGA database (https://tcga-data.nci.nih.gov/tcga/) [13]. In this study, MICAL1 mRNA expression and the association between its expression and the overall survival (OS), disease-specific survival (DSS), and progress-free interval (PFI) of patients with KIRC were analyzed in the TCGA-KIRC dataset. Based on the median values of mRNA expression, patients with KIRC were divided into high and low expression groups. Data were collected and statistically analyzed using R3.6.3 software [14].

### UALCAN

UALCAN is an interactive web portal to perform gene expression analyses using data from TCGA and protein expression analysis using data from Clinical Proteomic Tumor Analysis Consortium (CPTAC). We evaluated the expression of MICAL1 across KIRC and normal tissues by http://ualcan.path.uab.edu/ [15].

### Kaplan–Meier plotter database

The prognostic value of MICALs in KIRC was assessed according to OS using Kaplan–Meier plotter (https://kmplot.com/analysis/). Sources for this database includes the European Genome-phenome Archive (EGA), Gene Expression Omnibus (GEO), and TCGA [16].

### KEGG and GSEA

KEGG (https://www.genome.jp/kegg) is a collection of databases with genomes dealing with biological pathways, diseases, drugs, and chemical substances [17–19]. Differentially expressed genes (absolute fold change > 1.5, *p* adj <  0.05) were highly enriched. GSEA (https://www.gsea-msigdb.org/gsea) is a computational method that determines the classes of genes or proteins that are over-represented in a large set of genes or proteins and may have an association with disease phenotypes that are statistically significant [20, 21]. The normalized enrichment score was determined by analyzing 5000 permutations. *p* value < 0.05 and false discovery rate (FDR) <  0.25 were criteria to identify the significantly enriched gene set. KEGG and GSEA generated an ordered list of genes based on the correlation between all genes and MICAL1 expression. Data were collected and analyzed using the R3.6.3 software [14].

### Timer

TIMER (https://cistrome.shinyapps.io/timer/), a web server that provides analysis and visualization functions of tumor-infiltrating immune cells [22], was used to explore correlations between tumor-infiltrating immune cells and MICAL1.

### TISIDB

TISIDB (http://cis.hku.hk/TISIDB/index.php), a web portal that integrated multiple types of data resources in oncoimmunology [23], was used to investigate correlations between MICAL1 and immunoinhibitors.

### HPA

HPA (https://www.proteinatlas.org/), a web portal that aim to map all the human proteins in cells and tissues [24], was used to identify MICAL1 expression in KIRC.

### Cell lines

The Caki-1 and 786-O cell lines were purchased from the Cell Biology Institute of Chinese Academy of Science (Shanghai, China). Both cell lines were cultured at 37 °C in DMEM media, which contained 10% fetal bovine serum (Gibco, Carlsbad, USA) and 100 U/mL penicillin and 100 μg/mL streptomycin.

### siRNA transfection

The siRNAs were obtained from GenePharma (Shanghai, China). MICAL1 siRNA or control siRNA was transfected into cells according to the Lipofectamine 2000 DNA Transfection Reagent Protocol (Invitrogen). The siRNA-MICAL1 were as follows: #1, 5′-CAGCUUCUGUCACAGACAUTT-3′; #2, 5′-GACCUCCAGUGCUGUAUUATT-3′; #3, 5′-CAGGCACCAUGAAUAACUATT-3′.

### CCK8 assay

Cells were seeded into 96-well plates with 3 × 10^3^ cells/well and incubated for overnight. Then the cells were transfected with siRNA-MICAL1 or control siRNA. After incubated for the indicated time, 10 μL of CCK-8 (Bimake, Houston, Texas) were added to each well. After incubation in the dark at 37 °C for 40 min, the absorbance at 450 nm was obtained by a microplate reader (Bio-Tek, Elx800, USA).

### Wound healing assay

When the cells reached confluence in 6-well plates, a wound was made by scratching with 10 μl pipette tip. After rinsing with PBS, the cell monolayers were allowed to migrate for the indicated time. Photographs of wound spaces were taken using an inverted phase contrast microscope (Carl Zeiss Meditec, Jena, Germany).

### Transwell assay

Transwell assay was performed to evaluate cell migration, as we had reported previously [25]. 3 × 10^3^ cells were seeded in the upper chamber of a 24-well Transwell with 8 μm pore size. Medium with 10% FBS was added into the lower chamber. The assay was allowed to proceed at 37 °C for 48 h. A cotton swab was used to remove the cells on the upper side of membrane. Those cells that had moved to the lower surface of the membrane were stained with 0.1% crystal violet. Stained cells were scored in 5 random fields under an inverted microscope (Carl Zeiss Meditec, Jena, Germany).

### Western blotting

Western blotting assays were performed as described previously [9]. Antibodies targeting the following proteins were used: the polyclonal rabbit anti-human MICAL1 antibody (14818–1-AP, Proteintech, Wuhan, China), the polyclonal rabbit anti-human Rac1 antibody (24072–1-AP, Proteintech, Wuhan, China), the polyclonal rabbit anti-human GAPDH antibody (BS72410, Bioworld, Nanjing, China), the polyclonal goat anti-rabbit IgG (H + L) HRP (ZF0101, Zfanti, Nanjing, China). The enhanced chemiluminescence reagent solution was obtained from FuDeBio (HangZhou, China) and positive bands were analyzed using Quantity One (Bio-Rad, Hercules,USA). The images of all blots were provided in the Supplementary Information file, the blots were cut prior to hybridisation with antibodies.

### Pulldown assay

Active Rac1 was pulldown by PAK-CRIB beads as described previously [26]. Briefly, cells were lysed and protein in the supernatant was collected to new tubes containing beads precoupled with PAK-CRIB. After incubated under rotation at 4 °C for 30 min, the proteins bound on the beads were separated by SDS-PAGE and transferred to PVDF membrane. The amounts of active Rac1 were determined by corresponding antibodies and then visualized by the enhanced chemiluminescence reagent solution.

### Immunohistochemistry

Renal cancer tissue microarrays were purchased from Outdo biotech (Shanghai, China). Immunohistochemical staining was performed as described previously [27]. Briefly, microarray tissues were dewaxed and incubated MICAL1 (Proteintech, Wuhan, China) and secondary antibody (Maxim Biotechnologies, Fuzhou, China) overnight at 4 °C. Following incubation with HRP-labelled secondary antibody, tissue sections were stained with DAB under microscopic observation and counterstained with hematoxylin. Typical images were captured under Olympus BX51 microscope. By evaluating the percentage of the number of stained cells and the staining intensity of MICAL1, the immunoreactivity score (IRS) was evaluated as described previously [28–30]. IRS was calculated as intensity of the staining reaction (0 to 3 points) multiplied by the percentage of positive cells (0 to 4 points).

### Statistical analysis

All data were expressed as mean ± standard deviation. The chi-square test (the Fisher exact test was used when needed) was used for assess the correlations of clinicopathological parameters with MICAL1 expression. Kaplan-Meier method and Cox regression to evaluate the role of MICAL1 expression in prognosis. Statistically significant variables in univariate Cox regression were enrolled into multivariate Cox regression to validate the prognostic value of MICAL1. R statistical software was used to do bioinformatics analysis and build a prediction model. Spearman’s correlation coefficient was used to analyze the connection of MICAL1 with immunomodulators.

All calculations were performed with SPSS Version 20.0 (IBM Corp., Armonk, NY, USA). Statistical differences between two groups were tested using Student’s t-test. Comparisons among three or more groups were conducted using one-way ANOVA with a post-test to correct for multiple comparisons. *p* < 0.05 was taken to indicate statistical significance (two tailed).

## Results

### MICAL1 expression in patients with KIRC

**Fig. 1:**
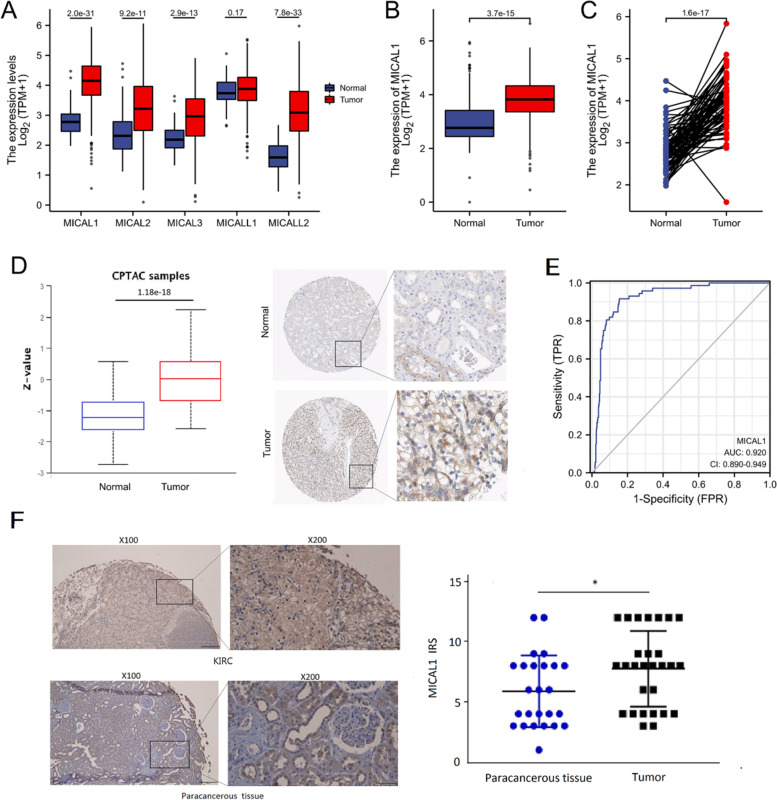
Expression of the MICAL family in KIRC and normal tissue. **a** Expression levels of MICALs in KIRC from the TCGA database. **b** The MICAL1 mRNA was dramatically more abundant in KIRC than non-paired normal tissues from the GTEx combined TCGA samples. **c** Differences in MICAL1 expression in KIRC samples and paired adjacent samples. **d** The MICAL1 protein expressions in KIRC compared with non-paired normal tissues. (E) ROC curve analysis evaluating the performance of MICAL1 for KIRC diagnosis. (F) Representative images of MICAL1 staining in KIRC and paracancerous tissues. *p < 0.05

To examine the function of MICALs in KIRC progression, the TCGA database was used to predict the mRNA expression pattern of each MICAL family member in the KIRC sample. MICAL1, 2, 3 and MICAL-L2 were significantly highly expressed in KIRC (*p *< 0.001, respectively) (Fig. 1a). Using Kaplan-Meier plotter analysis, MICAL1 and MICAL-L2 were further shown to be significantly correlated with poor OS in KIRC (Fig. S1). Since we chose MICAL1 as the focus of this paper, we further compared the expression of MICAL1 in normal samples of GTEx (28 cases) combined adjacent KIRC tissues (72 cases) and KIRC samples (531 cases) and found that MICAL1 was overexpressed in KIRC (*p *< 0.001) (Fig. 1b). Additionally, MICAL1 expression was significantly upregulated in 72 KIRC samples compared with that in matched adjacent samples (*p * < 0.001) (Fig. 1c). MICAL1 expression in various types of cancers was also investigated in the TCGA database (Fig. S2). Results available from the UALCAN (left) showed that the protein expression level of MICAL1 was upregulated in KIRC tissues (110 cases) in comparison with normal tissues (84 cases) (*p *< 0.001) (Fig. 1d), indicating that the mRNA and protein expressions of MICAL1 were similar in a different database. Representative images of MICAL1 expression in KIRC tissues and their normal controls downloaded from HPA (right) were also shown in Fig. 1d. The Receiving Operating Characteristic (ROC) curve analysis was applied to evaluate the diagnostic value of MICAL1 levels in KIRC patients. The area under the curve (AUC) value for MICAL1 levels was 0.920 (CI = 0.890*–*0.949) for detecting KIRC (Fig. 1e).

MICAL1 protein levels were also analyzed in a tissue microarray containing KIRC and paracancerous tissues. Although there is a few amount of dropping during the preparation of samples, the immunohistochemical analysis still showed that MICAL1 protein levels were significantly higher in KIRC tissues than in paracancerous normal tissues (Fig. 1f).

### The correlation between MICAL1 expression and clinical characteristics

**Fig. 2:**
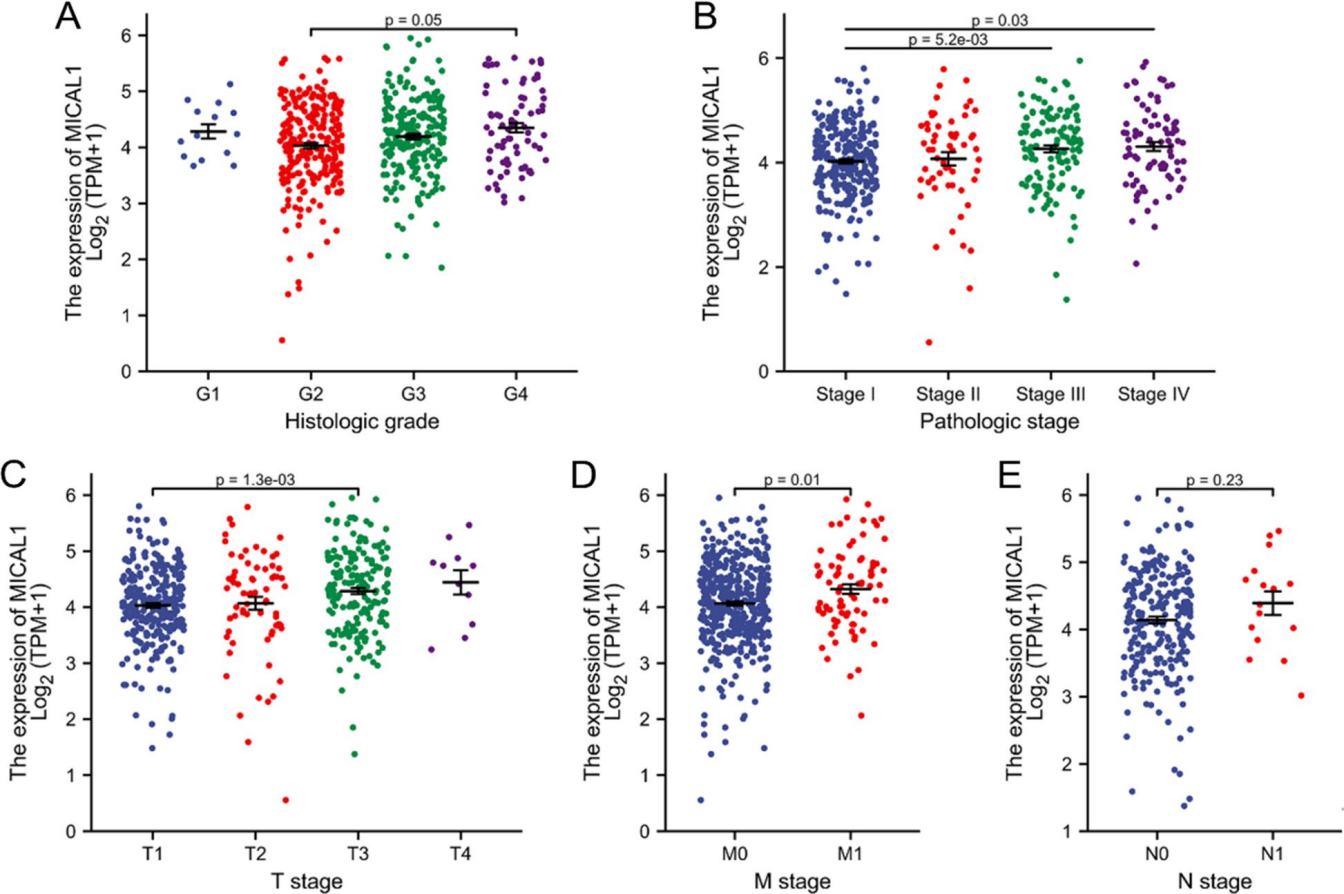
The relationship between MICAL1 expression and different clinical characteristics. **a** Histologic stage, **b **Pathologic stage, **c** T stage, **d** M stage, and **e** N stage

The characteristics of 530 patients with KIRC, including clinical and gene expression data, were collected from TCGA database. The patients were divided into high and low MICAL1 expression groups based on the mean value of MICAL1 expression (Table 1). To determine the significance of MICAL1 expression, putative correlation analysis between MICAL1 expression and clinical characteristics was performed. The results showed that MICAL1 mRNA expression differed significantly between grade 2 and grade 4 (grade 4 vs. grade 2, *p =* 0.05) as well as between stage I and stage III–IV tumors (stage III vs. stage I, *p =* 5.2e-03; stage IV vs. stage I, *p =* 0.03) (Fig. 2a and b). Higher expression of MICAL1 was also observed in T3 stage compared with T1 stage (*p =* 1.3e-03), M1 compared with M0 (*p =* 0.01), (Fig. 2c and d), but not N1 compared with N0 (*p =* 0.23) (Fig. 2e).

**Table 1:** The relationship between MICAL1 expression and clinicopathological data

Characteristic	Low expression of MICAL1	High expression of MICAL1	*p*
n	265	265	
T stage, n (%)			**< 0.001**
T1	158 (58.3%)	113 (41.7%)	
T2	32 (46.4%)	37 (53.6%)	
T3	72 (40.2%)	107 (59.8%)	
T4	3 (27.3%)	8 (72.7%)	
N stage, n (%)			0.572
N0	115 (48.1%)	124 (51.9%)	
N1	6 (37.5%)	10 (62.5%)	
M stage, n (%)			0.226
M0	223 (53.1%)	197 (46.9%)	
M1	35 (44.9%)	43 (55.1%)	
Pathologic stage, n (%)			**0.002**
Stage I	154 (58.1%)	111 (41.9%)	
Stage II	26 (45.6%)	31 (54.4%)	
Stage III	47 (38.2%)	76 (61.8%)	
Stage IV	37 (45.1%)	45 (54.9%)	
Primary therapy outcome, n (%)			0.610
PD	5 (45.5%)	6 (54.5%)	
SD	3 (60%)	2 (40%)	
PR	0 (0%)	2 (100%)	
CR	64 (53.3%)	56 (46.7%)	
Gender, n (%)			0.317
Female	87 (46.8%)	99 (53.2%)	
Male	178 (51.7%)	166 (48.3%)	
Race, n (%)			**0.021**
Asian	2 (25%)	6 (75%)	
Black or African American	20 (35.7%)	36 (64.3%)	
White	241 (52.5%)	218 (47.5%)	
Age, n (%)			0.193
< =60	124 (47%)	140 (53%)	
> 60	141 (53%)	125 (47%)	
Histologic grade, n (%)			0.268
G1	6 (42.9%)	8 (57.1%)	
G2	123 (54.2%)	104 (45.8%)	
G3	98 (47.6%)	108 (52.4%)	
G4	32 (42.7%)	43 (57.3%)	
Serum calcium, n (%)			0.142
Elevated	3 (30%)	7 (70%)	
Low	114 (56.2%)	89 (43.8%)	
Normal	73 (48.7%)	77 (51.3%)	
Hemoglobin, n (%)			0.291
Elevated	2 (40%)	3 (60%)	
Low	128 (49%)	133 (51%)	
Normal	103 (56%)	81 (44%)	
Laterality, n (%)			0.151
Left	116 (46.6%)	133 (53.4%)	
Right	149 (53.2%)	131 (46.8%)

To determine the prognostic value of MICAL1 in KIRC, a univariate logistic regression analysis was used to analyze the relationship between MICAL1 and clinical follow-up data in patients with KIRC (Table 2). A comparison of baseline data between the high and low expression groups revealed that MICAL1 expression was significantly associated with T stage (odds ratio [OR] = 1.986, *p* < 0.001), pathologic type (OR = 1.826, *p* < 0.001).

**Table 2:** Association of MICAL1 expression with clinicopathologic characteristics (logistic regression)

Characteristics	Total(N)	Odds Ratio(OR)	*p* value
Gender (Male vs. Female)	530	0.820 (0.573–1.171)	0.275
Age (> 60 vs. <=60)	530	0.785 (0.558–1.104)	0.165
T stage (T2-T4 vs. T1)	530	1.986 (1.408–2.812)	***
N stage (N1 vs. N0)	255	1.546 (0.556–4.669)	0.413
M stage (M1 vs. M0)	498	1.391 (0.857–2.270)	0.183
Pathologic stage (Stage III- IV vs. Stage I- II)	527	1.826 (1.283–2.609)	***
Histologic grade (G3-G4 vs. G1-G2)	522	1.338 (0.948–1.891)	0.098
Primary therapy outcome (CR vs. PD/SD/PR)	138	0.700 (0.251–1.895)	0.483

***: *p* < 0.001

### The independent diagnostic value of MICAL1 expression in KIRC

**Fig. 3:**
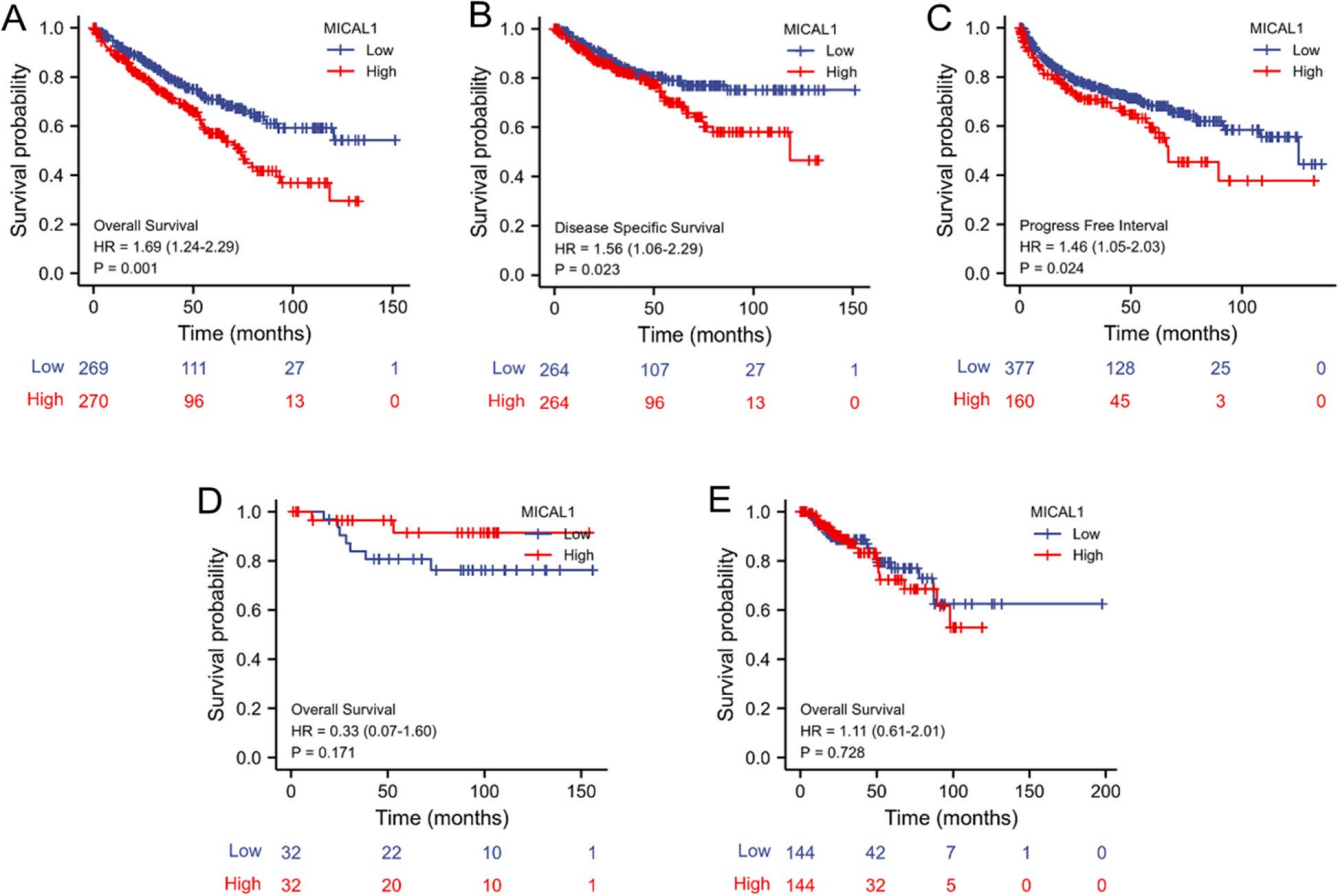
Kaplan-Meier analysis. **a** OS, **b** DSS, and **c** PFI in patients with KIRC between high- and low-MICAL1 expression groups. OS in patients with **d** KICH and **e** KIRP between high- and low-MICAL1 expression groups

The survival analysis demonstrated that high MICAL1 expression was correlated with poor OS (*p =* 0.001) as well as poor DSS (*p =* 0.023) when the patients were split by median (Fig. 3a and b). High MICAL1 expression was also correlated with poor PFI (*p =* 0.024) when using the auto-select best cutoff option (Fig. 3c). Unlike the results obtained in KIRC, high expression of MICAL1 was not significantly correlated with poor OS in the other two kinds of renal carcinoma, KICH (*p =* 0.171) and KIRP (*p =* 0.728) (Fig. 3d and e).

**Fig. 4:**
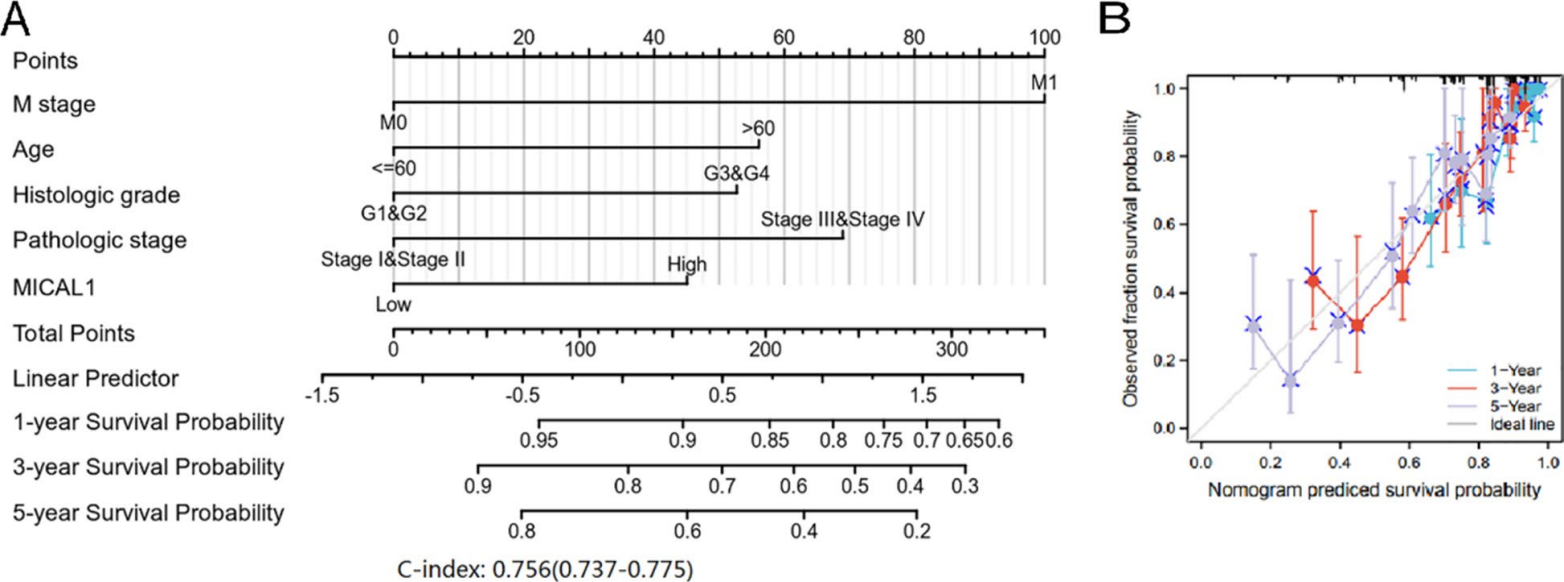
Prediction model of nomogram construction. **a** A nomogram for predicting the probability of 1-, 3- and 5-year OS in KIRC patients. **b** Calibration plots validating the efficiency of nomograms for OS

Univariate and multivariate analyses were performed to determine whether high expression of MICAL1 was an independent risk factor for poor OS in patients with KIRC. Univariate analysis showed that T stage, M stage, age, histologic grade, pathologic stage, and MICAL1 expression were the factors influencing OS. Cox multivariate analysis showed that MICAL1 expression was an independent risk factor for tumor progression (*p =* 0.007). Simultaneously, the M stage, age, histologic grade, and pathologic stage also affected tumor progression (Table 3). Next, the independent prognostic factors of the Cox regression analysis were integrated to construct the prognostic nomogram to predict 1-, 3-, and 5-year OS in patients with KIRC (Fig. 4a). In the internal validation, the c-index of the nomogram was 0.756 (95% CI: 0.737–0.775). The bias-corrected line in the calibration plot was close to the ideal curve, indicating a desirable prediction of the nomograms (Fig. 4b). These data suggest that elevated MICAL1 expression significantly shortens OS in patients with KIRC. MICAL1 may be a valuable biomarker for the prediction of KIRC.

**Table 3:** Univariate and multivariate Cox proportional hazards analysis of MICAL1 expression and OS for patients with KIRC

Characteristics	Total (N)	Univariate analysis		Multivariate analysis	
		Hazard ratio (95% CI)	*p* value	Hazard ratio (95% CI)	*p* value
T stage (T2-T4 vs. T1)	530	2.872 (2.063–3.998)	***	0.753 (0.402–1.409)	0.375
M stage (M1 vs. M0)	498	4.333 (3.170–5.922)	***	2.532 (1.737–3.689)	***
Age (> 60 vs. <=60)	530	1.753 (1.290–2.383)	***	1.685 (1.231–2.306)	**
Gender (Male vs. Female)	530	1.052 (0.771–1.434)	0.750		
Histologic grade (G3-G4 vs. G1-G2)	522	2.660 (1.888–3.748)	***	1.677 (1.156–2.434)	**
Pathologic stage (Stage III- IV vs. Stage I- II)	527	3.860 (2.809–5.305)	***	2.354 (1.254–4.418)	**
MICAL1 (High vs. Low)	530	1.686 (1.243–2.287)	***	1.536 (1.124–2.100)	**

**: *p* < 0.01, ***: *p* < 0.001

### MICAL1 enhances the migration of renal clear cell carcinoma cells

**Fig. 5:**
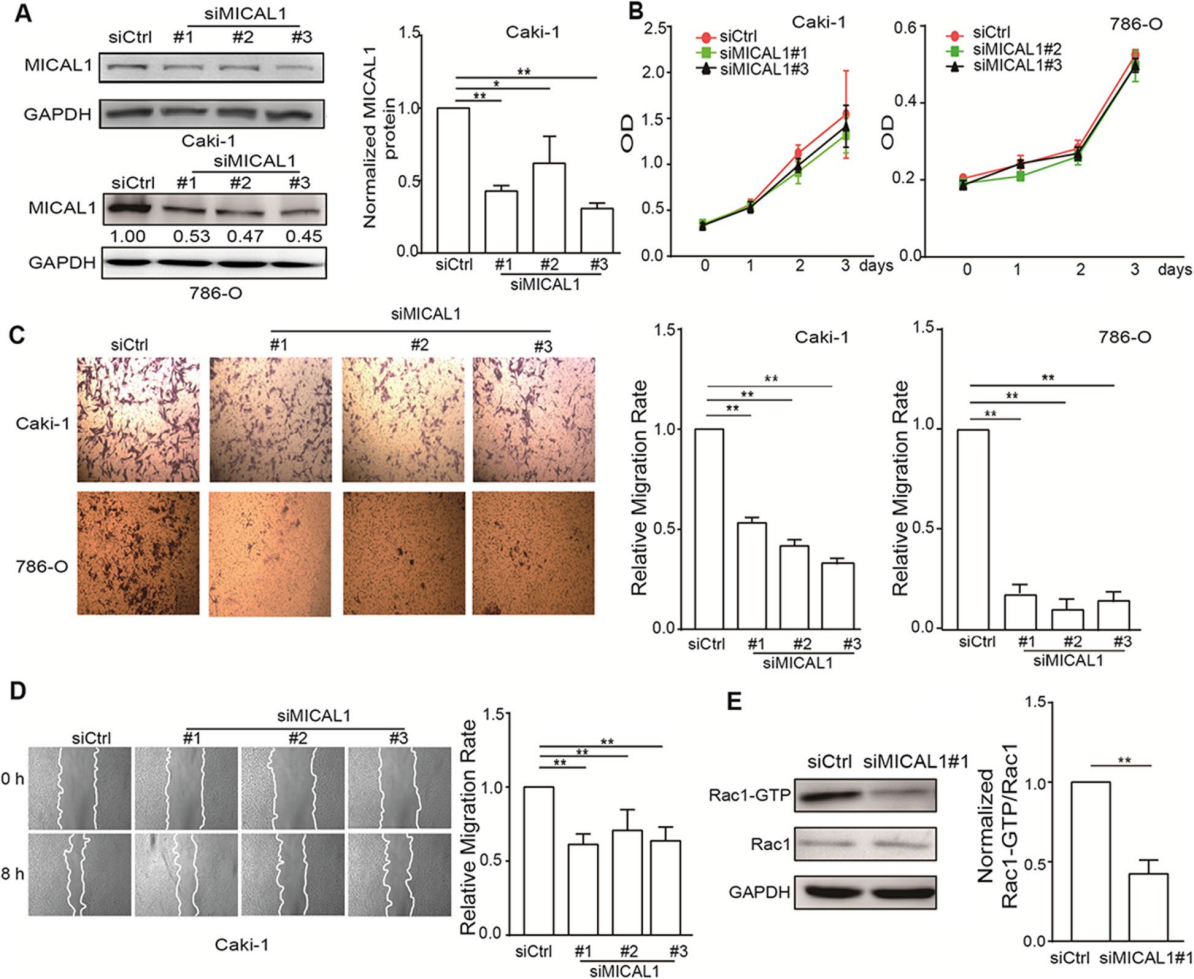
The effect of MICAL1 on Rac1 activation and renal clear cell carcinoma cell migration. **a** Caki-1 and 786-O cells were transfected with control siRNA or siRNA specifically targeting MICAL1 (siMICAL1) for 48 h, then total protein extracts from those cells were analyzed for MICAL1 expression. Western blot bands corresponding to MICAL1 were quantified and normalized against GAPDH. The statistical graph from Caki-1 cells was shown in Fig. 1A panel on the right. **b** After knockdown of MICAL1 for 48 h, the proliferation rates of Caki-1 and 786-O cells were assessed by CCK8 assays. (C&D) After knockdown of MICAL1 for 36 h, the migratory rates of these cells were assessed by Transwell **c** and wound healing assays **d**. **e** Levels of GTP-Rac1 in MICAL1-knockdown Caki-1 cells were assessed. *p < 0.05, **p < 0.01

To analyze the role of MICAL1 in the progression of renal clear cell carcinoma, we performed MICAL1 loss-of-function assays in renal clear cell carcinoma cells. First, we silenced MICAL1 expression in Caki-1 and 786-O cells using siMICAL1. The knockdown efficiency was determined by western blotting (Fig. 5a). As shown in ​Fig. 5b and d, transfection with siMICAL2 failed to inhibit proliferative ability of those cells (​Fig. 5b), but effectively impaired migratory potential of those cells, as measured by Transwell and wound healing assays (Fig. 5c and d). Transfection with siMICAL1 in Caki-1 cells also led to a significant reduction in Rac1 activation (GTP form of Rac1) (Fig. 5e), which has been proposed to have pro-migratory effects in most types of cancer cells [31]. Full length blots of Fig. 5a and e were included in Fig. S3. Together, the results suggested the role of MICAL1 in promoting migratory ability of renal clear cell carcinoma cells.

### Function enrichment of MICAL1 in KIRC

**Fig. 6:**
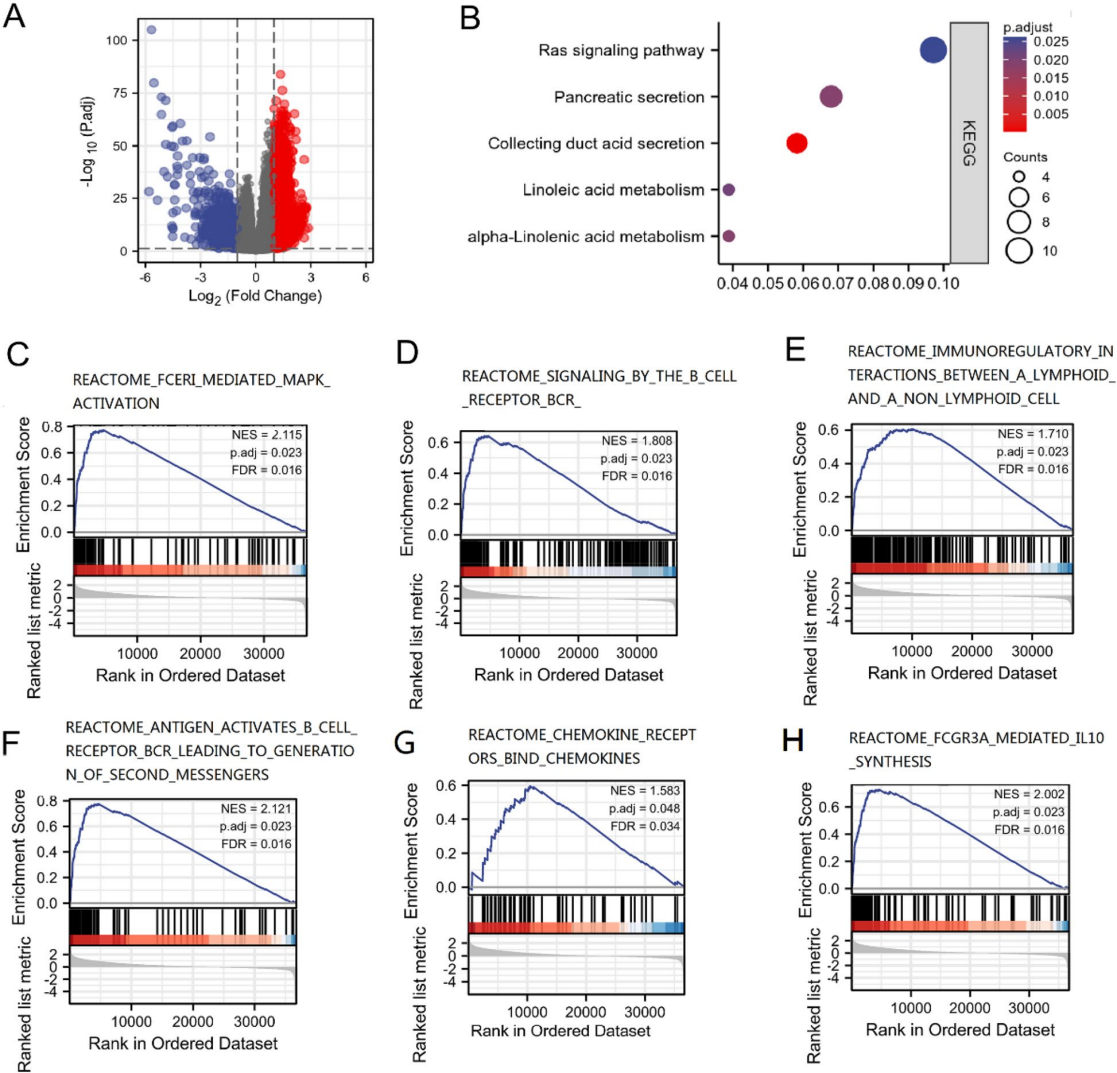
Functional enrichment analysis of MICAL1 in KIRC. **a** Volcano plot of differentially expressed genes between the high and low MICAL1 expression groups. **b** KEGG pathway analysis with high MICAL1 expression. **c–h** Enrichment plots of MICAL1-relevant enrichment pathways in c2.cp.v7.2.symbols.gn in KIRC obtained after GSEA analysis

In order to better understand the functional implication of MICAL1 in KIRC, we explored the possible cellular mechanism through KEGG and GSEA. As shown in Fig. 6a, differentially expressed genes (DEGs) (|logFC| > 1, adjusted *p*-value < 0.05) between high- and low-MICAL1 groups were identified, including 5588 upregulated and 505 downregulated genes. Figure 6b shows that hsa04014 (Ras signaling pathway) is the most relevant enrichment pathway in the high MICAL1 group. The components hsa04966 (Collecting duct acid secretion), hsa00592 (alpha-Linolenic acid metabolism), hsa04972 (Pancreatic secretion), and hsa00591 (Linoleic acid metabolism) were also associated with the functions of MICAL1 in KIRC. We also found that the Ras downstream pathway, MAPK, was one of the most relevant enrichment pathways in the high MICAL1 group (Fig. 6c). GSEA analysis showed that MICAL1 was related to immunoregulatory interactions, reactome signaling by the B cell receptor, and IL10 synthesis processes (Fig. 6d and h). Since a large part of the functional annotation and predicted signaling pathways were related to an immune reaction, we hope to explore immune infiltration further to better explain the function of MICAL1 in KIRC.

### The association between MICAL1 and immune infiltration

**Fig. 7:**
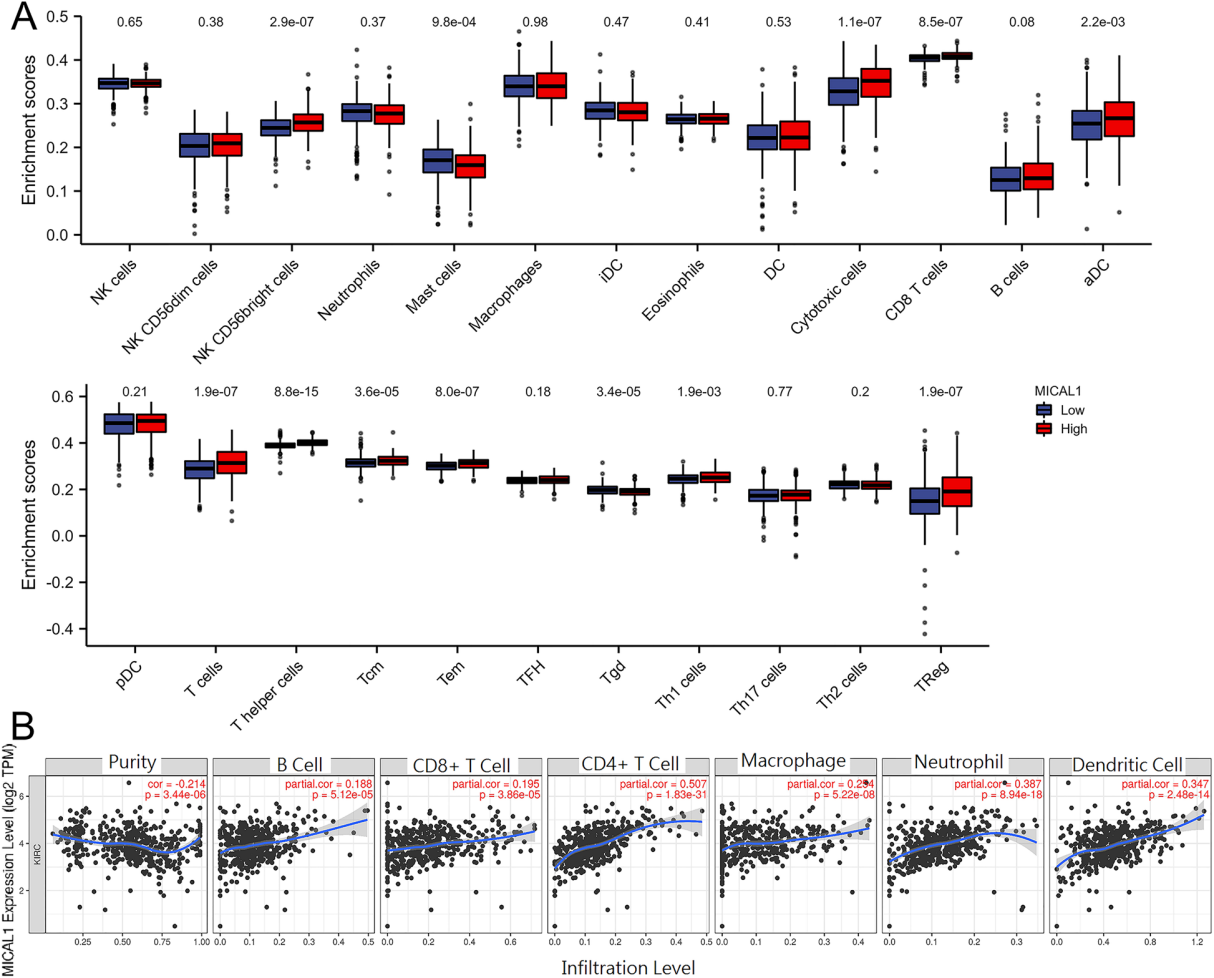
The correlation of MICAL1 and immune infiltration in KIRC. **a** A comparison of immune cells with high or low expression of MICAL1. **b** Correlations between MICAL1 expression and immune infiltration levels in KIRC by TIMER. NK, natural killer; DC, dendritic cell; iDCs, immature DC; aDC, activated DC; pDC, plasmacytoid DC; Tcm, T central memory; Tem, T effector memory; TFH, T follicular helper; Tgd, T gamma delta; Th, T helper cells; Th1, type 1 Th cells; Th2, type 2 Th cells; Th17, type 17 Th cells; Treg, regulatory T cells

Then, we explored the association between MICAL1 expression and immune cell infiltration level quantified by ssGSEA in KIRC using Spearman correlation. MICAL1 was positively associated with the abundance of NK CD56bright cells (*p* < 0.001), cytotoxic cells (*p *< 0.001), CD8 T cells (*p *< 0.001), aDC cells (*p =* 2.2e-03), T cells (*p *< 0.001), T helper cells (*p *< 0.001), Tcm cells (*p *< 0.001), Tem cells (*p *< 0.001), Th1 cells (*p =* 1.9e-03), Treg cells (*p *< 0.001) in cell infiltration levels. In the meantime, it was negtively associated with the abundance of Mast cells (*p *< 0.001), Tgd cells (*p *< 0.001). By TIMER analysis, B cells (partial.cor = 0.188), CD4+ cells (partial.cor = 0.507), CD8+ cells (partial.cor = 0.195), neutrophils (partial.cor = 0.387), dendritic cells (partial.cor = 0.347), and macrophage (partial.cor = 0.254) infiltration levels were positively associated with MICAL1 expression significantly (Fig. 7a and b). The analyses above indicate that MICAL1 expression is correlated with immune infiltration level, especially with CD8+ T cells, in KIRC.

**Fig. 8:**
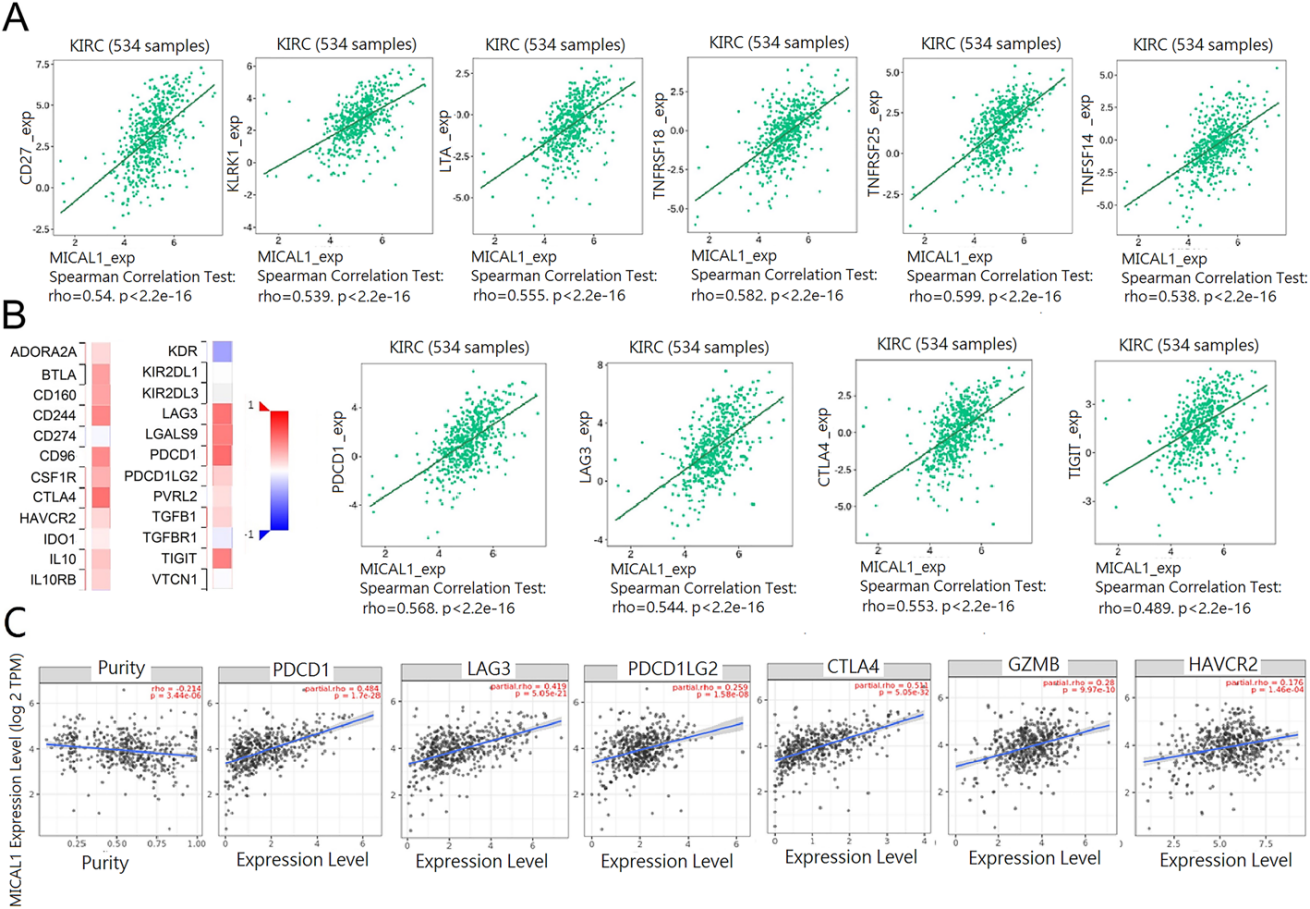
The correlation of MICAL1 and immune immunomodulators in KIRC. **a** Correlations between MICAL1 and immune immunostimulators in KIRC at TISIDB database. **b** Correlations between MICAL1 and immune inhibitors in KIRC. **c** Correlations between MICAL1 and gene markers of T cell exhaustion in KIRC by TIMER

To further confirm the relationship between MICAL1 expression and immune cell infiltration levels in KIRC, we used TISIDB to explore the correlations between MICAL1 expression and various immunomodulators. Our results showed a significant correlation between MICAL1 expression and immunostimulators, including CD27 (rho = 0.54), PLRK1 (rho = 0.539), LTA (rho = 0.555), TNFRSF18 (rho = 0.582), TNFRSF25 (rho = 0.599), TNFSF14 (rho = 0.538) (Fig. 8a). In addition to the expression of immunostimulators, the expression of MICAL1 was also specifically closely associated with immunoinhibitors PDCD1 (rho = 0.568), LAG3 (rho = 0.544), CTLA4 (rho = 0.553), and TIGIT (rho = 0.489) (Fig. 8b). Furthermore, the results form TIMER implicated that PDCD1 (partial.rho = 0.484), LAG3(partial.rho = 0.419), PDCD1G2 (partial.rho = 0.259), CTLA4 (partial.rho = 0.511), GZMB (partial.rho = 0.28), HAVCR2 (partial.rho = 0.176) of T-cell exhaustion, are all strongly correlated with MICAL1 in KIRC (Fig. 8c). We also assessed the connection of MICAL1 with the aforementioned markers of T-cell exhaustion according to GEPIA database, and the correlations are similar with that in TIMER (data not shown). Therefore, the results demonstrated MICAL1 was closely correlated with immune infiltration including T cell exhaustion markers in KIRC.

## Discussion

While only one gene encodes MICAL in *Drosophila*, vertebrates contain five genes encoding MICAL isoforms (*MICAL1, 2, 3*) and MICAL-like isoforms (*MICAL-L1, −L2*). MICALs are not only widely expressed in the nervous system [32], their abnormal expressions have been found in multiple types of tumors, including gastric, breast, lung, and prostate cancer [33–35]. Some members of this family were identified as carcinogenic genes and positively correlated with the more severe tumor prognosis. For example, the high expression of MICAL-L2 was related to tumor immunity and tumor progression of renal clear cell carcinoma. High cytoplasmic MICAL2 and/or total MICAL2 expression levels were also identified as positively correlated with lymphatic metastasis and shorter OS of lung adenocarcinoma patients [35]. Among these isoforms, MICAL1 has the most closely related domain architecture to *Drosophila* MICAL [36]. Recent study suggested that *MICAL1* gene disruption could altered breast cancer cell cytoskeleton organization, cell morphology and inhibited cell migration [37], however, only a few reports have been published to describe the functions of MICAL1 during cancer progression.

In our study, we found that both MICAL1 mRNA and protein expressions were significantly upregulated in KIRC tissues compared with normal tissues. In addition, higher MICAL1 expression implied advanced pathologic stage, histologic grade, T stage, and M stage for KIRC. While low expression of MICAL1 was found in colorectal cancer (CRC), and silencing of MICAL1 promoted CRC cell migration and enhanced EMT [38], the results from bioinformatic analysis strongly suggested that MICAL1 was a cancer-promoting gene in KIRC. We further investigated the prognostic value of MICAL1 in KIRC using the Kaplan-Meier Plotter, which showed that patients with high MICAL1 expression have worse OS, DSS than those with low MICAL1 expression. The results of univariate and multivariate analysis showed that high MICAL1 expression was an independent risk factor for poor OS in individuals with KIRC. ROC analysis also confirmed the diagnostic value of MICAL1. The in vitro study showed that knockdown of MICAL1 led to a decreased Rac1 activation and migration of clear cell carcinoma cells. Collectively, these results indicated that the expression of MICAL1 may predict the prognosis and promising therapeutic target of KIRC.

To explore more molecular mechanisms of MICAL1 in KIRC, we conducted the GSEA and KEGG tools to carry out data mining for KIRC based on gene expression datasets from TCGA. GSEA and KEGG analysis revealed the function of MICAL1 enriched in the Ras signaling pathway and immune response in KIRC. It is well known that constitutively active Ras is the most common oncogene in human cancer. Normally, Ras operates two main cellular pathways: mitogen-activated protein kinases (MAPK) and phosphoinositide-3 kinase (PI3K) pathways. The subfamilies of MAPK contain extracellular signal-regulated kinase (ERK), c-Jun N-terminal kinase (JNK), and p38 MAPK. Activation of ROS-sensitive ERK/cyclin D pathway is an essential mechanism mediating breast cancer cell proliferation by MICAL1 [9]. Consistently, overexpression of MICAL1 augmented the generation of ROS, activated PI3K/Akt signalling, and favored invasive phenotypes of breast cancer cells [10]. Although KIRC is a malignant tumor of urinary tubular epithelial system and has complex biological characteristics, our data implied that MICAL1 might play a critical role in KIRC carcinogenesis and progression through similar mechanisms.

KIRC has long been identified as a highly immune-infiltrated tumor [39]. It was reported that a higher proportion of regulatory T (Treg) cells was associated with a worse outcome in KIRC [40]. CD8+ T cells are preferred to enhance ferroptosis-specific lipid peroxidation and increase ferroptosis, contributing to the anti-tumour efficacy of immunotherapy [41]. Treg cells, cancer-associated fibroblasts and macrophage type 2 cells could make immunologic barriers against CD8+ T cell-mediated antitumor immune responses [42]. However, recent study showed the abundance of intratumoral infiltration of CD8+ T cells, which has the ability to secrete CXCL13, indicated inferior clinical outcome in patients with KIRC [43]. Increasing evidence has revealed that renal cell carcinoma displays large expansion of double positive CD4+ CD8+ T Cells with expression of exhaustion markers [44]. Notably, in this study, MICAL1 expression was found positively correlated with CD8+ T and Tregs infiltrating levels, as well as T cell exhaustion markers such as PDCD1, LAG3 and CTLA4.

Programmed cell death protein 1 (PD1, encoded by PDCD1), which is known to negatively regulate T-cell activation, is expressed in multiple kinds of T cells, including CD4+, CD8+, Treg and NK. In addition, the immune inhibitor molecules CTLA4 and LAG3 were associated with a poor prognosis in KIRC [40]. CTLA4 can be found in Treg cells [45]. LAG3 (+) Treg cells were also found to suppress macrophages’ proinflammatory activation in colorectal cancer patients [46]. Antibody drugs against immune checkpoint proteins, including PD-L1, PD-1 and CTLA4, are FDA approved for treating a wide range of cancers [47]. Since PD1, CTLA4, and LAG3 expressions are closely related to CD8+ T and Treg cells, we speculated that MICAL1 might promote the development and progression of KIRC through regulating the function of CD8+ T and/or Treg cell. In order to accurately understand the relationship between MICAL1 and immune infiltration in KIRC, further investigation is needed to verify the results.

There were inadequacies in the present study. The data analyzed was mainly based on the online databases. We only checked the migratory and proliferative abilities of renal clear cell carcinoma cells after silencing MICAL1 in those cells. These in vitro experiments were too preliminary to depict the potential functions of MICAL1 in renal clear cell carcinoma. Extensive functional in vitro and in vivo work is needed to validate these predictions.

## Conclusion

Together, this analysis revealed that MICAL1 was highly expressed in KIRC and its high expression level correlated with poor patient outcome. MICAL1 could be considered a prognostic factor for KIRC patients, which might activate immune infiltration, Ras signaling and cell migration. Although the current study, for the first time, reveals the potential function between MICAL1 and immune infiltration, the issue about how MICAL1 precisely regulates KIRC progression is likely to be settled in further studies.

## Supplementary Information

**Additional file 1: Fig. S1.** Kaplan-Meier analysis for OS in patients with KIRC between high- and low-MICALs expression groups. Fig. S2. mRNA expression of MICAL1 in different tumor types from TCGA database. (*p < 0.05, **p < 0.01, ***p < 0.001). Fig. S3. Blots in Fig. 5a and e were shown. Figure 5a (up), Fig. 5a (down), Fig. 5e

## Data Availability

Availability of data and materials from TCGA dataset (https://tcga-data.nci.nih.gov/tcga/).

## Abbreviations

AUC: area under the curve
CC: coiled-coil
CH: calponin homology
CI: confidence interval
DSS: disease specific survival
ERK: extracellular signal-regulated kinase
FAD: flavin adenine dinucleotide
GTEx: genotype-tissue expression
GEO: gene expression omnibus
GSEA: gene set enrichment analysis
HPA: human protein atlas database
HR: hazard ratio
JNK: c-Jun N-terminal kinase
KEGG: kyoto encyclopedia of genes and genomes
KIRC: kidney renal clear cell carcinoma
KIRP: kidney renal papillary cell carcinoma
KICH: kidney chromophobe
LIM: Lin11,Isl-1 and Mec-3
IRS: immunoreactivity score
MAPK: mitogen-activated protein kinases
MICAL: molecules interacting with casL
NCBI: national center for biotechnology information
NES: normalized enrichment score
OR: odds ratio
OS: overall survival
PD1: programmed cell death protein 1
PFI: progression free interval
PI3K: phosphoinositide-3 kinase
ROC: receiver operating characteristic
TCGA: the cancer genome atlas
Treg: regulatory T
