# Supplementary material for: High MICAL1 expression correlates with cancer progression and immune infiltration in renal clear cell carcinoma
Source: BMC Cancer. 2022 Dec 27;22:1355. doi: 10.1186/s12885-022-10462-1 (PMC9793553; doi:10.1186/s12885-022-10462-1)
Supplement: Supplementary file 1 — Additional file 1: Fig. S1. Kaplan-Meier analysis for OS in patients with KIRC between high- and low-MICALs expression groups. Fig. S2. mRNA expression of MICAL1 in different tumor types from TCGA database. (*p < 0.05, **p < 0.01, ***p < 0.001). Fig. S3. Blots in Fig. 5a and e were shown. Figure 5a (up), Fig. 5a (down), Fig. 5e [file 12885_2022_10462_MOESM1_ESM.docx]

**Figure S1. Kaplan-Meier analysis for OS in patients with KIRC** **between high- and low-MICALs expression groups.**


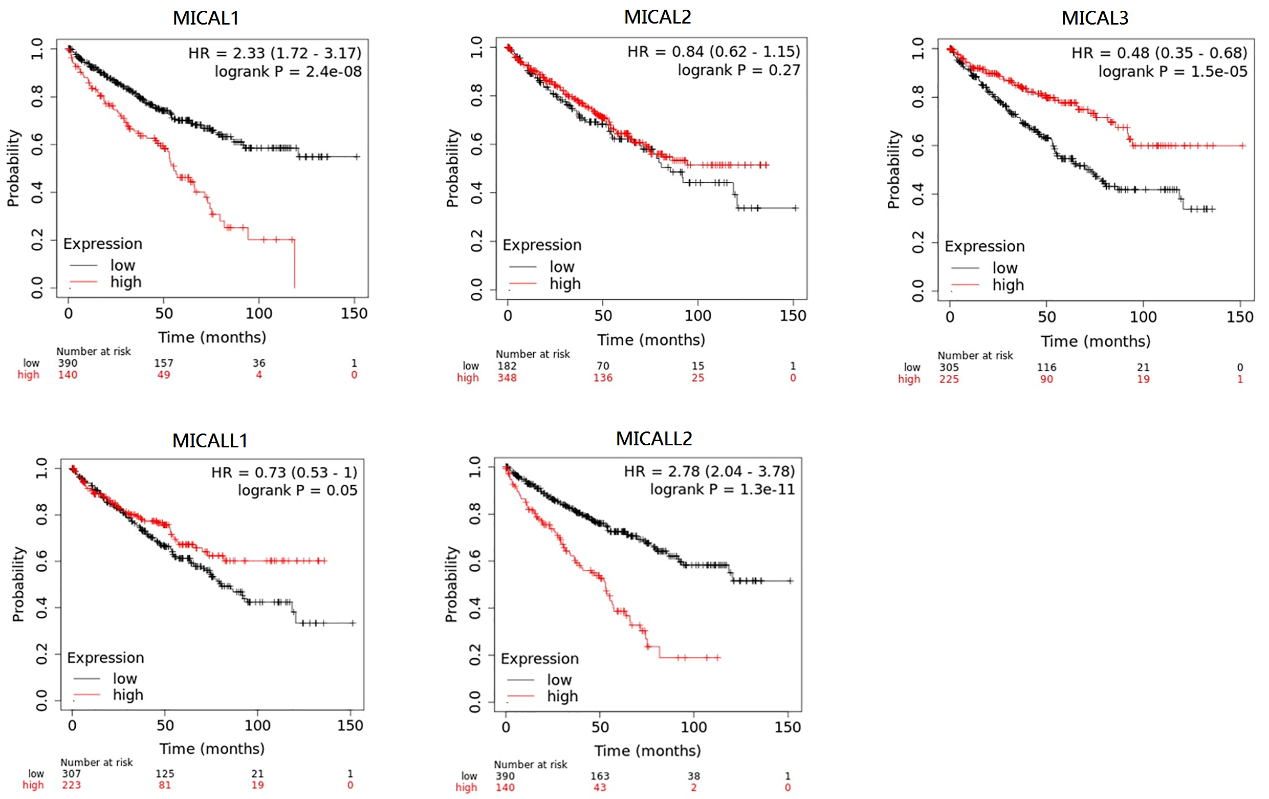


**Figure S2. mRNA expression of MICAL1 in different tumor types from TCGA database.** (**p* < 0.05, ***p* < 0.01, ****p* < 0.001)


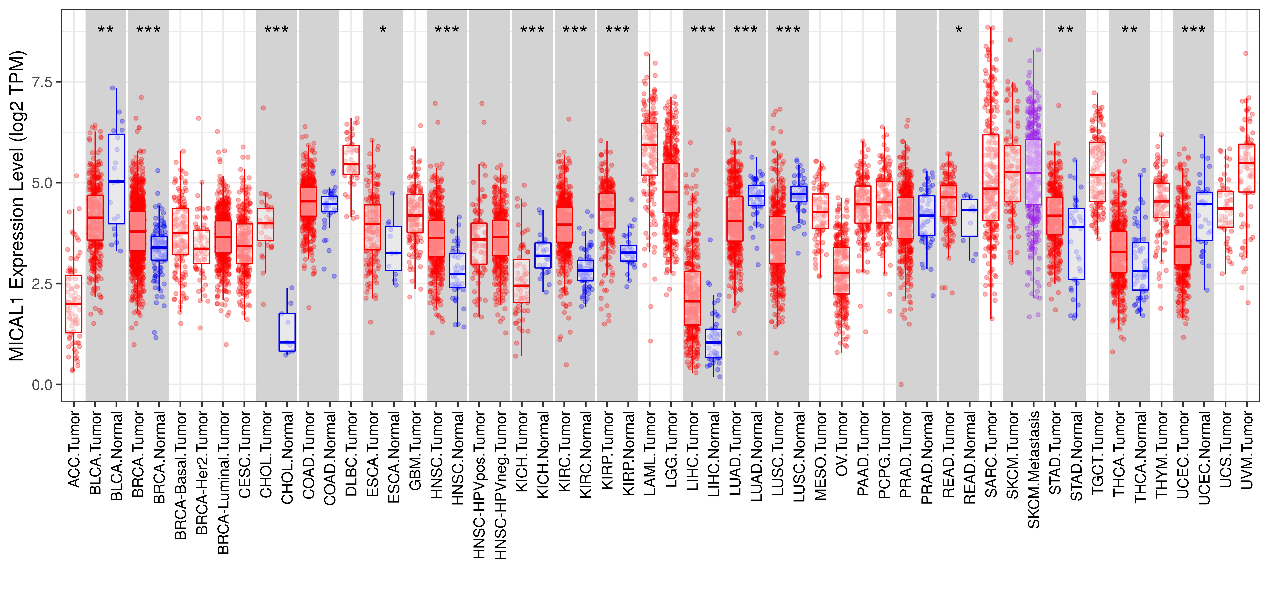


**Figure S3. Blots in Fig. 5A&5E were shown.**

**Fig.5A (up)：**


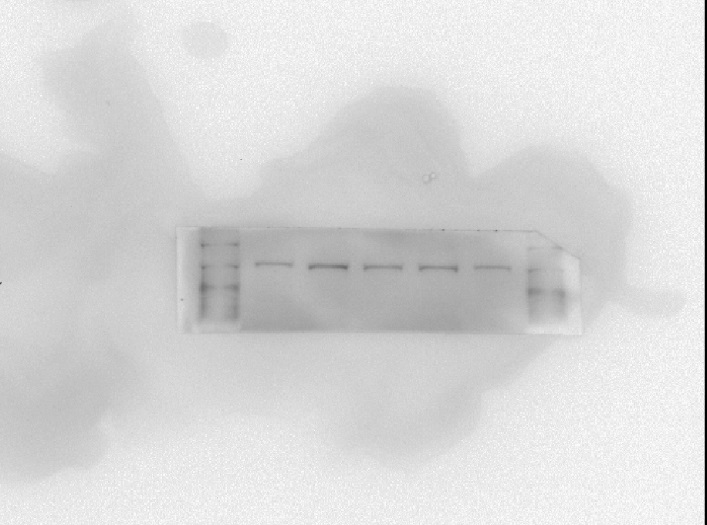


siC 1 2 3

siRNA

MICAL1

130


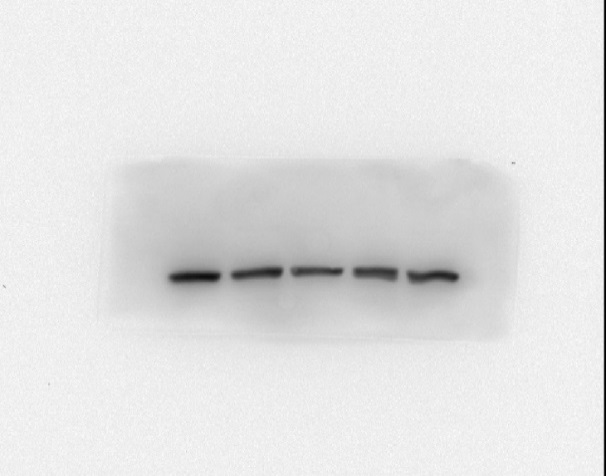


GAPDH

siRNA

siC 1 2 3

36

**Fig. 5A (down):**

130

MICAL1

siRNA

siC 1 2 3








GAPDH

36

siC 1 2 3

siRNA

**Fig. 5E**





siC siMICAL1

Rac1-GTP

21





siC siMICAL1

Rac1

21





siC siMICAL1

GAPDH

36
